# Genome-Wide In Silico Analysis of 1-Aminocyclopropane-1-carboxylate oxidase (ACO) Gene Family in Rice (*Oryza sativa* L.)

**DOI:** 10.3390/plants13243490

**Published:** 2024-12-13

**Authors:** Jing Xia, Yingsheng Qiu, Wanli Li, Yingcheng Zhang, Linxin Liu, Yi Wang, Wangshu Mou, Dawei Xue

**Affiliations:** College of Life and Environmental Sciences, Hangzhou Normal University, Hangzhou 311121, China; 2022111010015@stu.hznu.edu.cn (J.X.); 2023111010033@stu.hznu.edu.cn (Y.Q.); 2022210301199@stu.hznu.edu.cn (W.L.); 2023111010025@stu.hznu.edu.cn (Y.Z.); 2021210315162@stu.hznu.edu.cn (L.L.); 2021210315124@stu.hznu.edu.cn (Y.W.)

**Keywords:** 1-Aminocyclopropane-1-carboxylic acid oxidase (ACO), ethylene biosynthesis, rice, transcriptional regulation, post-translational modification, abiotic stress

## Abstract

The plant hormone ethylene elicits crucial regulatory effects on plant growth, development, and stress resistance. As the enzyme that catalyzes the final step of ethylene biosynthesis, 1-Aminocyclopropane-1-carboxylic acid oxidase (ACO) plays a key role in precisely controlling ethylene production. However, the functional characterization of the *ACO* gene family in rice remains largely unexplored. In this study, we performed a phylogenetic analysis of seven *OsACO* genes (*OsACO1*–*OsACO7*), which were classified into three subfamilies (Types I, II, and III). The members within the same clades exhibited similar tertiary structures and conserved protein motifs. We conducted inter/intraspecies covariance assays of OsACOs to elucidate their evolutionary and duplication events. Numerous cis-acting elements identified in *OsACO* promoter regions are associated with development, hormonal stimuli, and environmental responses. The expression assay by RT-qPCR revealed that *OsACO* genes exhibited tissue-specific expression and were significantly altered under various abiotic stresses, indicating their potential involvement in these processes regulated at the transcriptional level. Additionally, we predicted candidate-targeting miRNAs and identified putative cysteine sites of S-nitrosylation (SNO) and S-sulfhydration (SSH) in OsACOs, providing insights into their post-transcriptional and post-translational regulatory mechanisms. These findings pave the way for the further investigation of OsACO functions and their potential applications in improving rice growth and stress resilience by modulating ethylene biosynthesis.

## 1. Introduction

Ethylene, a gaseous plant hormone, plays a vital role in regulating diverse biological processes including plant growth and development, senescence, leaf abscission, fruit ripening, and responses to environmental stresses [1]. In higher plants, ethylene is synthesized starting with the conversion of S-adenosyl-methionine (AdoMet) to 1-Aminocyclopropane-1-carboxylic acid (ACC) by ACC synthase (ACS), followed by the oxidation of ACC to ethylene by ACC oxidase (ACO) [2]. While ACS is widely regarded as the rate-limiting enzyme [3], accumulating evidence suggests that ACO plays pivotal roles for ethylene production during certain conditions, such as the post-climacteric ripening of tomatoes [4], pea seed germination [5], and fiber cell elongation in cotton [6]. Therefore, ACO is under a tight regulatory mechanism at the multiple levels of transcription, translation, and protein stability or activity, contributing to the precise control of ethylene biosynthesis.

The ACO enzyme belongs to the 2-oxoglutarate-dependent dioxygenase (2OGD) subfamily, which is classified into the DOXC53 clade [7]. Unlike other 2OGD enzymes, ACO uniquely utilizes ascorbate acid, instead of 2OG, as a reductant and co-substrate with molecular oxygen [7]. All 2OGDs are characterized by a double-stranded β-helix fold (DSBH) containing a conserved motif (His-Xaa-Asp/Glu-(Xaa)n-His), which is responsible for the ligation of ferrous iron Fe (II) [7,8]. The ligated iron thus serves as a cofactor essential for binding the carboxylate of ACC into ACO enzymes [9,10,11]. In addition, bicarbonate is required as an activator for catalyzing the oxidation of ACC to ethylene [8].

Rice (*Oryza sativa* L.) is one of the world’s most important food crops, with its yield influenced by an array of developmental, hormonal, and environmental factors. The modulation of ethylene production plays a critical role in controlling these processes. Tao et al. (2025) reported that increased endogenous ethylene promotes the formation of root endodermal barrier, reducing the radical transport and accumulation of cadmium in rice (Nipponbare) [12]. Ethylene has been reported to promote rice seedling establishment (including seed germination, the elongation of the coleoptile/mesocotyl, and root growth, etc.), contributing to the determination of agronomic traits [13]. Zhang et al. (2022) identified a very-long-chain fatty acid synthesis gene, *SD38*, as a regulator of plant height by activating ethylene biosynthesis in rice (Jinhui10), highlighting the significance of ethylene in crop breeding [14].

ACO enzymes, which catalyze the final step in ethylene biosynthesis, are crucially involved in their regulation in rice [15]. *OsACO1*, the first ACO member cloned from rice (Pin Gaew 56 and Habiganj Aman II), showed significantly induced expression and enzyme activity in submerged deepwater rice, suggesting that ethylene production is modulated, at least partially by ACO, to promote internode elongation under submergence [16]. Chae et al. (2000) isolated the cDNA clones of *OsACO2* and *OsACO3* in rice plants (Japonica) and found that their expressions were regulated by intricate crosstalk between auxin and ethylene [17]. Iwai et al. (2006) observed that ethylene biosynthesis, rather than ethylene signaling, is essential for resistance to blast fungus in young rice plants (Nipponbare), primarily contributed by the induced expression of *OsACO5* and *OsACO7* [18]. However, most studies on OsACOs have mainly demonstrated transcriptional alterations while their detailed functions and regulatory mechanisms remain largely unexplored.

In the present study, we performed a comprehensive genome-wide analysis of seven members of the *OsACO* gene family, examining phylogenetic relationships, collinearity, chromosomal locations, protein structures, motif patterns, promoter cis-elements, predicted targeting miRNA, putative post-translational modification sites, and gene expression patterns in different tissues and stresses. These analyses aimed to advance our understanding of the *OsACO* gene family, shedding light on its roles in ethylene biosynthesis. Our findings provide a foundation for the future functional verification of OsACOs and valuable insights into potential applications for improving rice growth, stress resilience, and yield through the regulation of ethylene production via modulating OsACOs’ functions.

## 2. Results

### 2.1. Phylogenetic Analysis of the OsACO Gene Family

**Figure 1:**
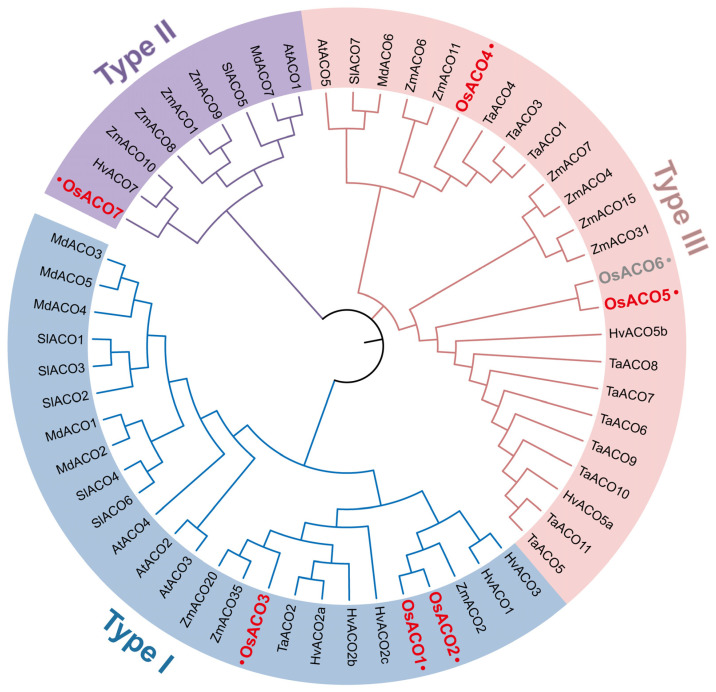
Phylogenetic analysis of the *ACO* gene family across multiple plant species. The phylogenetic tree was conducted including *Oryza sativa* (Os), *Arabidopsis thaliana* (At), *Solanum lycopersicum* (Sl), *Zea mays* (Zm), *Hordeum vulgare* (Hv), *Malus domestica* (Md), and *Triticum aestivum* (Ta), using the neighbor-joining method with 1000 bootstrap replicates (MEAG11). Different colors indicate the three subfamilies (Types I, II, and III) of *ACO* gene family. *OsACO* genes were marked in red while *OsACO6*, a predicted pseudogene, was marked in gray.

To elucidate the phylogenetic relationships of the rice *OsACO* gene family, we performed a cluster analysis of *ACO* homologs from six annotated species, including Arabidopsis, wheat, barley, maize, tomato, and apple (Figure 1). The results grouped all *ACO* genes into three subfamilies. Based on the established classification of AtACOs in Arabidopsis [19], we also categorized the seven OsACOs into three clades (Types I, II, and III). Specifically, OsACO1 (LOC_Os09g27820.1), OsACO2 (LOC_Os09g27750.1), and OsACO3 (LOC_Os02g53180.2) were assigned to Type I; OsACO7 (LOC_Os01g39860.1) to Type II; and OsACO4 (LOC_Os11g08380.1), OsACO5 (LOC_Os05g05680.1), and OsACO6 (LOC_Os05g05670.1) to Type III. Among them, Iwai et al. (2006) identified OsACO6 as a pseudogene encoding a truncated ACO protein through sequence alignment [18].

### 2.2. Interspecific Collinearity Analysis of OsACO Gene Family

**Figure 2:**
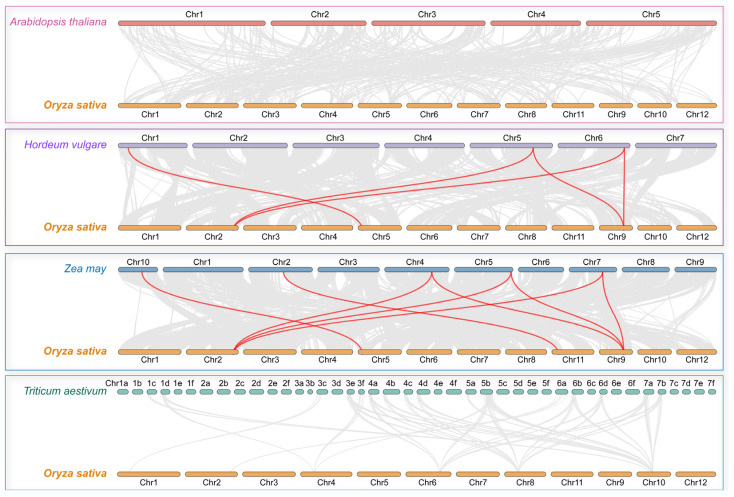
Interspecific collinearity analysis of *ACO* genes of rice with other plant species. The analysis was conducted with rice (*Oryza sative*), *Arabidopsis thaliana*, barley (*Hordeum vulgare*), maize (*Zea mays*), and wheat (*Triticum aestivum*). Grey lines in the background represent collinear blocks between rice and other species while red curves specifically indicate the syntenic *ACO* gene pairs.

To investigate the evolution of *OsACO* gene family members, we performed comparative collinearity analyses of rice with other representative species, including Arabidopsis, barley, maize, and wheat. Our findings revealed no significant collinearity between rice *OsACO*s and those of Arabidopsis or wheat. In contrast, we identified collinearity with barley and maize, where three rice *OsACO* genes corresponded to five homologous gene pairs in barley, and four aligned with eight pairs in maize (Figure 2). Notably, *OsACO2* on Chr9 and *OsACO3* on Chr2 exhibited high homology with the genes in both barley and maize, suggesting their strong conservation and potential important roles during the evolutionary processes of these cereal crops.

To evaluate the evolutionary pressure on *OsACO*s, we calculated the Ka/Ks ratios for these syntenic gene pairs. All the estimated values ranged from 0.22 to 0.48 (Supplementary Table S1), which were far below 1.0, suggesting that the evolution of *OsACO*s was primarily under purifying selection to conserve their functional stability.

### 2.3. Chromosomal Distribution and Duplication Event Analysis of the OsACO Gene Family

**Figure 3:**
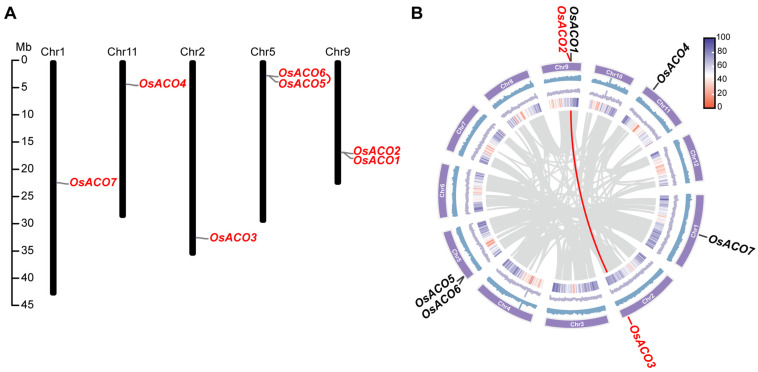
Chromosomal distribution and duplication events of rice *OsACO* genes. (**A**) Chromosomal (Chr) distribution of *OsACO* family members in rice, with chromosomes drawn to scale based on their actual physical lengths (Mb). The red linker indicates the tandem duplication. (**B**) Localization and segmental duplication events of *OsACO* genes in the rice genome. The red line represents intraspecific collinearity.

Chromosomal localization analysis revealed that seven *OsACO* genes are unevenly distributed across five chromosomes. Specifically, *OsACO7* is localized on chromosome 1, *OsACO4* on chromosome 11, *OsACO3* on chromosome 2, *OsACO5* and *OsACO6* on chromosome 5, and *OsACO1* and *OsACO2* on chromosome 9 (Figure 3A). Notably, *OsACO5* and *OsACO6* form a tandem duplication cluster, as determined by the McScanX program in TBtools (Figure 3A). We found that the mRNA sequence of *OsACO6* is highly similar to that of *OsACO5*, except for mutations in the 5′-UTR that result in a delayed translational start and a mutation in the coding sequence (from TAC to TAA) that introduces a premature stop codon. We hypothesized that *OsACO6*, duplicated from *OsACO5*, may function as a pseudogene encoding a truncated protein. In addition, the analysis showed that *OsACO2* and *OsACO3* were collinear with each other (Figure 3B). These results suggest that both tandem and segmental duplications may have contributed to the expansion of the *OsACO* gene family.

### 2.4. OsACO Protein Structure Analysis

The secondary structures of the OsACO proteins were predicted using the SOPMA online tool (https://npsa-pbil.ibcp.fr/cgi-bin/npsa_automat.pl?page=npsa_sopma.html (accessed on 8 June 2024)). The results showed that OsACO proteins were primarily composed of random coils (35.67–42.86%), α-helices (32.48–39.88%), extended strands (16.20–21.66%), and β-turns (5.45–10.19%) (Table 1, Supplementary Table S2). Among them, random coils and α-helices were the predominant elements in the secondary structures of OsACO proteins.

**Figure 4:**
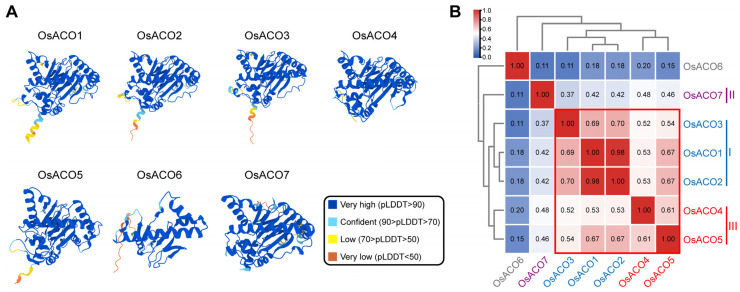
Tertiary structure analysis of OsACO proteins. (**A**) The predicted 3D structures of OsACO proteins using AlphaFold2. The accuracy of protein modeling was indicated by the score of pLDDT (predicted Local Distance Difference Test) ranging from 0 to 100. (**B**) Topological similarity of OsACOs assessed using the TM-score. The scores higher than 0.5 assumed a generally similar structural fold between the compared protein pairs (highlighted in the red box).

We then utilized the AlphaFold2 online platform (https://alphafold.ebi.ac.uk/ (accessed on 18 September 2024)) to predict the 3D structures of OsACO proteins (Figure 4A). Tertiary structural similarities among the OsACO family members were assessed by using the TM-score tool (http://zhanggroup.org/TM-score/ (accessed on 12 September 2024)). OsACO6 is a truncated protein, showing the greatest divergence from all the OsACOs (Figure 4A,B). The results revealed that OsACO1, OsACO2, OsACO3, OsACO4, and OsACO5 shared higher similarity (all pairwise TM-scores above 0.5) while OsACO7 displayed distinct structural features compared with the other members (Figure 4B). These findings were consistent with their subfamily classification based on protein sequence similarity.

### 2.5. Analysis of Protein Conserved Motifs in OsACO Proteins

**Figure 5:**
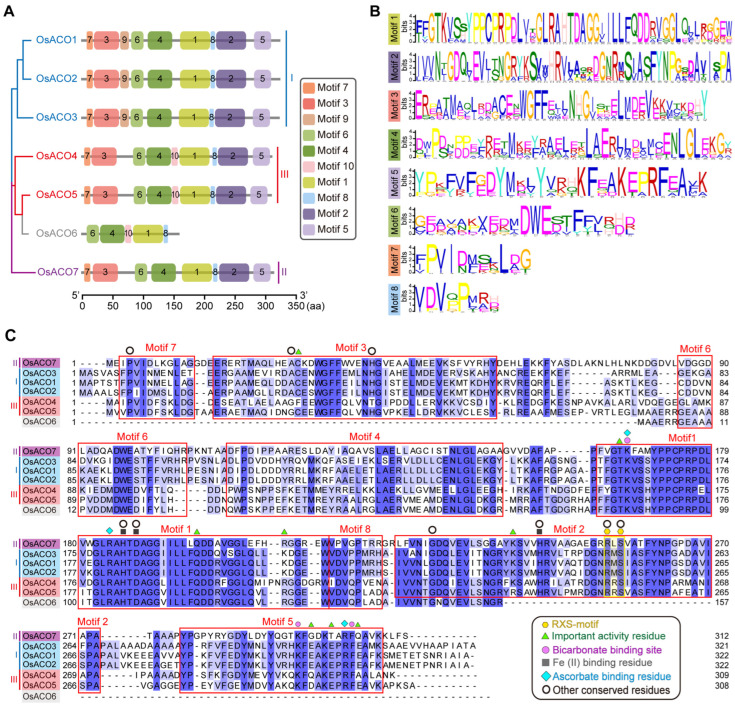
The conserved motifs of OsACO proteins in rice. (**A**) Distribution diagram of conserved motifs across OsACOs, with different colored rectangles representing distinct motifs. (**B**) Sequences of top eight enriched motifs identified by MEME. (**C**) Protein sequence alignment performed by Jalview, with conserved motifs 1–8 highlighted with red boxes and key residues indicated by different symbols.

We analyzed the motifs of the OsACO family using the MEME online website (https://meme-suite.org/meme/tools/meme (accessed on 17 August 2024)) and identified ten motifs, named from motif 1 to 10 based on their degrees of conservation (Figure 5A,B and Supplementary Table S3). As expected, closely related OsACO members in the phylogenetic tree shared common motifs, implying functional similarity within clades (Figure 5A). Specifically, motifs 1–8 were conserved across all the OsACOs while motif 9 was specific to Type I and motif 10 to Type III, and both motifs were absent in Type II (Figure 5A). OsACO6, as a truncated protein, lacks most conserved motifs found in other family members (Figure 5A).

We also aligned the predicted protein sequences of OsACOs and confirmed the presence of the same conserved motifs (motif 1–8) identified by MEME (Figure 5C). Based on the key residues reported in other plant species (summarized in Houben et al. [19]), we marked the conserved sites of OsACOs that may be critical for the ACO enzyme activity in ethylene production (Figure 5C). The Fe (II) binding sites, consisting of two conserved His residues and a carboxylate group (from Asp or Glu), coordinate Fe (II) as a metal cofactor to facilitate the binding of ACC to the ACO enzymes [9,10,11]. These sites are located within motifs 1 and 2, which are well conserved across all OsACOs (indicated by black rectangles in Figure 5C). The RXS (Arg-X-Ser) motif, responsible for binding the carboxylate group of ACC [20], is highly conserved throughout the entire *OsACO* gene family (except in OsACO6) (yellow hexagons in Figure 5C). The three subfamilies of OsACOs exhibit variations in the intermediate residue within the RXS motif: R-M-S for Type I (OsACO1/2/3), R-L-S for Type II (OsACO7), and R-R-S for Type III (OsACO4/5) (Figure 5C).

Other essential residues involved in ascorbate and bicarbonate binding, as well as those proven to be critical for enzyme activity, are positioned throughout motifs 1, 2, 3, and 5 (Figure 5C). OsACO7 lacks three important activity residues in motif 5 (equivalent to E299, E302, and E306 in OsACO1) while OsACO4 lacks two (E299 and E306), and OsACO5 lacks one (E299) (Figure 5C, indicated by green triangles). The ACO enzymes are members of the Fe (II)/ascorbate-dependent dioxygenase family, which share nine highly conserved residues (indicated by black circles in Figure 5C) [21]. OsACO4 and OsACO5 were found to be missing several of these conserved residues within motif 3 (A30 and H42 for OsACO4, and A30 for OsACO5) (Figure 5C). Therefore, whether these absent residues render the corresponding OsACO proteins nonfunctional remains to be experimentally verified.

### 2.6. Analysis of Cis-Acting Elements in the OsACO Gene Promoters

**Figure 6:**
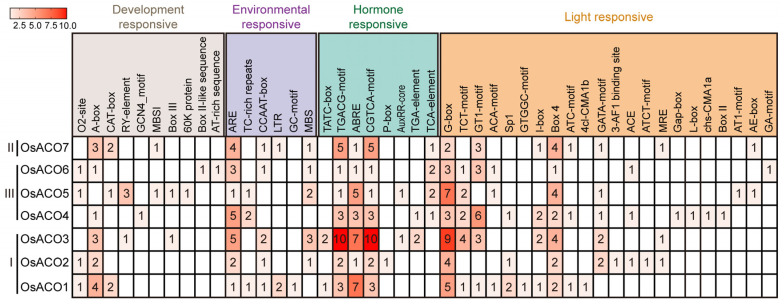
Cis-elements analysis in the promoter sequences of *OsACO* genes. The putative cis-elements classified by their established functions were quantified with the numbers shown in the squares.

To further explore the putative transcriptional regulatory mechanisms and biological functions of the *OsACO* gene family, we analyzed the genomic sequence ranging from 2559 bp to 4439 bp upstream of the gene start codon (ATG) by using the PlantCare online tool (http://bioinformatics.psb.ugent.be/webtools/plantcare/html (accessed on 16 September 2024)). The predicted cis-acting elements in the promoter regions are illustrated in Figure 6. Our analysis identified several cis-acting elements associated with various biological processes, including growth and development and responses to environmental stress, hormones, and light (Figure 6, Supplementary Table S4).

The results revealed ten cis-acting elements related to growth and development, such as CAT-box, MBSI, the GCN4 motif, and the RY-element, which may function in meristematic tissue expression, flavonoid synthesis, endosperm expression, and seed-specific regulation. Additionally, six elements are associated with abiotic stress, such as ARE, the GC motif, TC-rich repeats, LTR, and MBS, which are implicated in responses to hypoxia, low temperature, and drought stress. Eight elements related to hormonal regulation were identified, including TATC-box and P-box (GA response), the TGACG motif and CGTCA motif (MeJA response), ABRE (ABA response), AuxRR-core and the TGA element (auxin response), and the TCA element (SA response). Additionally, twenty-two light responsive elements were targeted, with G-box, the GT1 motif, and Box 4 occurring most frequently. Overall, the promoter regions of OsACOs contained a higher number of cis-elements responsive to both hormone stimuli and environmental stresses, suggesting that the significance of OsACOs in ethylene biosynthesis may promote their potential involvement in hormone crosstalk and stress resilience.

### 2.7. miRNA Targeting Sites Prediction

**Figure 7:**
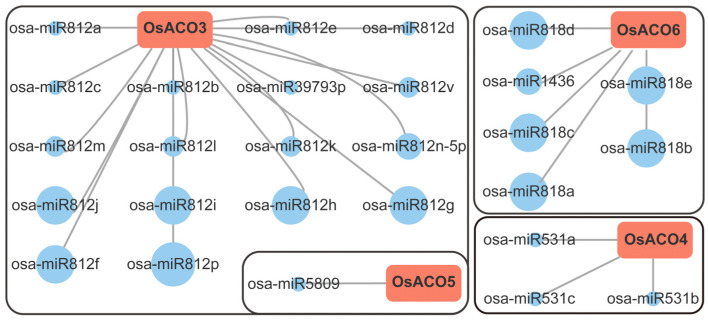
Prediction of miRNA-targeting *OsACO* genes in rice. Red rectangles indicate OsACO family members and blue solid circles denote the putative targeting miRNAs. Circle size of each miRNA inversely correlates with the expectation values determined by psRNATarget, with larger circles indicating higher possibility of target accessibility.

MicroRNAs (miRNAs), 21–24 nucleotide (nt) short small non-coding RNAs, regulate gene expression at the post-transcriptional level by targeting complementary mRNAs for cleavage or translational repression, playing critical roles in diverse plant developmental processes [22]. Using the plant small-RNA target server psRNATarget (https://www.zhaolab.org/psRNATarget/analysis?function=2 (accessed on 20 September 2024)) with default parameters (expectation values set to ≤ 3.0), we identified a total of 27 miRNAs targeting four out of seven *OsACO* genes, including *OsACO3*, *OsACO6*, *OsACO4*, and *OsACO5* (Figure 7, Supplementary Table S5). *OsACO3* was predicted to be targeted by the maximum number of miRNAs while *OsACO5* was targeted by only one miRNA. Each miRNA is specific to its corresponding *OsACO* gene, with the cleavage identified as the primary regulatory effect (Figure 7, Supplementary Table S5).

The possibility for the miRNA targeting of *OsACO* genes, evaluated by expectation values from psRNATarget (Supplementary Table S5), is represented by a positive correlation with circle sizes in Figure 7. Osa-miR812p, osa-miR812f, and osa-miR812g are the top targets for *OsACO3*, osa-miR531a and osa-miR531b target *OsACO4*, and osa-miR5809 is the main target for *OsACO5* (Figure 7).

### 2.8. Putative Post-Translational Modifications of OsACOs

**Figure 8:**
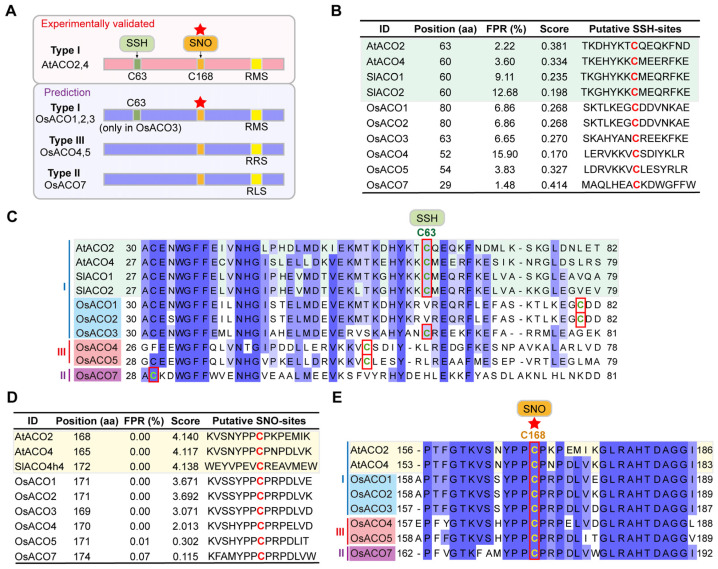
Prediction of S-sulfhydration (SSH) and S-nitrosylation (SNO) modification sites in OsACOs. (**A**) Diagram illustrating the validated and predicted cysteine sites for both SSH and SNO modifications. (**B**) The FDR, scores, and detailed SSH sites predicted by pCysMod. (**C**) Alignment of ACO protein sequences from rice, Arabidopsis, and tomato. The validated or putative SSH cysteine sites have been marked with red rectangles. (**D**) The FDR, scores, and the detailed SNO sites predicted by pCysMod. (**E**) Alignment of ACO protein sequences from rice and Arabidopsis. The validated or putative SNO cysteine sites have been indicated by red rectangles. The experimentally verified SSH sites in ACOs from Arabidopsis and tomato have been highlighted with green background for (**B**,**C**), and SNO sites in yellow for (**D**,**E**). The red stars shown in (**A**–**E**) indicate the well-conserved cysteine sites (equivalent to C168 in AtACO2) for SNO modification across AtACO2/4 and all OsACOs.

Post-translational modifications (PTMs) influence various aspects of protein functionality, including stabilization, degradation, enzyme activity, subcellular localization, and interactions [23]. While ACO enzymes serve as the final key step in ethylene biosynthesis, their involved PTM mechanism remains largely unknown. Some redox-based modifications of ACO proteins have been reported in other plant species. In tomato, S-sulfhydration (SSH) was identified at Cys60 in both SlACO1 and SlACO2, which led to the inhibition of ACO enzyme activity and reduced ethylene production [24]. Additionally, proteomic analysis in Arabidopsis detected the persulfidation of AtACO2 and AtACO4, with C63 in AtACO2 and C60 in AtACO4 highly conserved to C60 in SlACO1 and SlACO2 [25] (Figure 8A–C). Given that the PTM mechanisms of OsACOs in rice are yet to be elucidated, we aimed to investigate the potential SSH sites within OsACOs. Several putative cysteine residues for SSH were identified in the OsACO family using pCysMod (http://pcysmod.omicsbio.info/ (accessed on 20 September 2024)) (Supplementary Table S6). For each OsACO, we selected the top candidate site with the lowest false positive rate (FPR) and highest score and compared them with the experimentally validated sites from the literature (Figure 8B,C). We found these candidates showed comparable FDR and score values to the validated sites (Figure 8B), but only C63 in OsACO3 was well conserved at its position when aligned with the reported cysteine sites in Arabidopsis and tomato (Figure 8A,C). These findings provided directions for the further experimental confirmation of SSH modifications in OsACO proteins.

Another type of cysteine modification, S-nitrosylation (SNO), has been observed in AtACO2 (C168) and AtACO4 (C165) [26]. In tomato, SlACO homolog 4 (SlACOh4) was S-nitrosylated at C172, resulting in its enzyme activation and improved ethylene synthesis [27,28]. We found that all OsACOs possess a highly conserved cysteine site (as C171 in OsACO1), consistent with those reported in Arabidopsis and tomato (equivalent to C168 in AtACO2) (Figure 8A,E, Supplementary Table S6). Furthermore, OsACO1 to OsACO4 exhibited similar FPR levels (0.00%) and scores (from 2.013 to 3.671) to those validated sites whereas OsACO5 and OsACO7 showed significantly lower scores (0.302 and 0.115, respectively) (Figure 8D). Additional evidence is required to determine whether this conserved cysteine site across all OsACOs can undergo S-nitrosylation and its specific effects on OsACO functions.

### 2.9. Expression Patterns of OsACOs in Different Tissues

**Figure 9:**
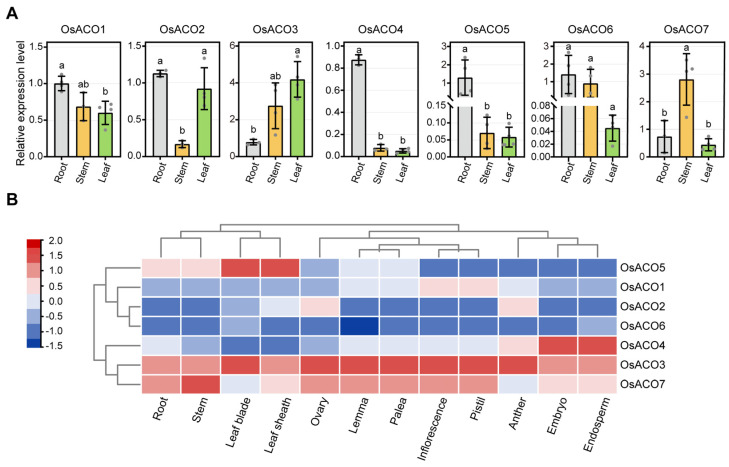
Expression pattern analysis of the *OsACO*s in different rice tissues. (**A**) Expression level analysis of *OsACO*s in the root, stem, and leaf in wild-type seedlings at 14 days after gemination by qRT-PCR. Data represent the means ± S.Ds. n = 3 to 4 for each sample. The expression level in the root for each gene was normalized to 1.0. Different letters indicate significant differences determined by one-way ANOVA with Tukey’s test. (**B**) Expression profiles of *OsACO*s in different tissues of wide type during the reproductive stages, obtained from RiceXPro database. The color scale represents relative expression levels.

We next examined the expression patterns of *OsACO* genes in different tissues of wild-type seedlings during early vegetative growth (14-day-old seedlings), including in root, stem, and leaf, by using qRT-PCR (Figure 9A). We found that *OsACO1* and *OsACO6* showed similar expression level across all three organs while *OsACO2* was mainly expressed in the root and leaf and *OsACO3* primarily in the stem and leaf (Figure 9A). *OsACO4* and *OsACO5* were predominantly expressed in the root, with extremely low levels in the stem and leaf, whereas *OsACO7* was highly expressed in the stem (Figure 9A). These findings suggest that *OsACO*s exhibited tissue-specific expression and may play distinct roles in plant development.

Furthermore, we obtained the transcript profiles of the *OsACO* gene family in various tissues during the reproductive stages of rice from the RiceXPro database (https://ricexpro.dna.affrc.go.jp/ (accessed on 13 June 2024)) and generated heatmaps using TBtools (Figure 9B). The results showed that *OsACO3* and *OsACO7* were constitutively expressed across all the tissues during the reproductive stages while *OsACO2* and *OsACO6* exhibited generally lower expression levels (Figure 9B). *OsACO4* was highly expressed in the endosperm and embryo whereas its expression in the stem and leaf was relatively low (Figure 9B), consistent with the RT-qPCR results observed in the earlier developmental stages (Figure 9A). In contrast, *OsACO5* displayed predominant expression in the leaf sheath and blade, which differed from the RT-qPCR results during the vegetative stages (Figure 9B). This discrepancy was probably due to the distinct transcriptional regulation of *OsACO5* at different developmental stages.

### 2.10. Expression Profiles of OsACOs Under Abiotic Stresses

**Figure 10:**
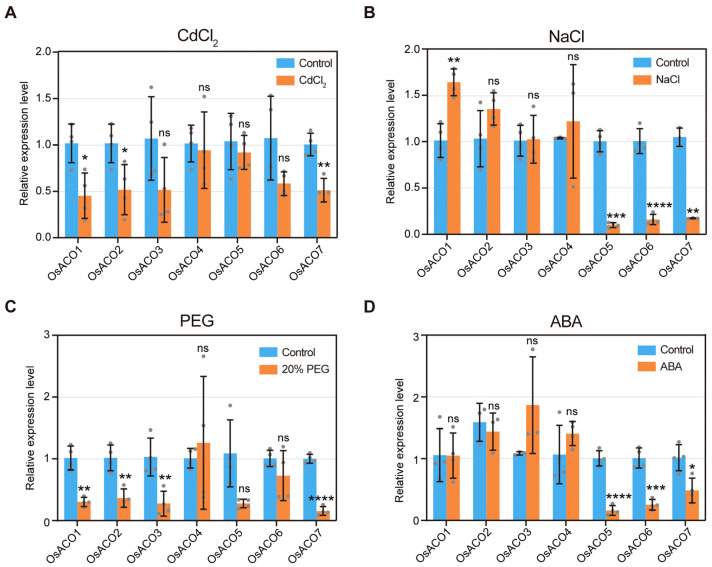
Expression levels of *OsACO*s in 14-day-old wild-type seedlings after exposure to different abiotic stresses or ABA for 24 h. (**A**) Treatment with 50 μM CdCl_2_. (**B**) Treatment with 150 mM NaCl. (**C**) Treatment with 20% PEG 6000. (**D**) Treatment with 50 μM ABA. Data are presented as means ± S.D.s (n = 3 to 4 per sample). For all treatments, expression levels in the control group for each gene were normalized to 1.0. Asterisks indicate significant differences between the control and treated group (* *p* < 0.05, ** *p* < 0.01, *** *p* < 0.001, and **** *p* < 0.001; Student’s *t*-test) and ns represents no significant difference.

Given the presence of putative cis-acting elements related to environmental stress and hormonal stimuli in the promoter regions of *OsACO* genes, we would like to examine their transcriptional responses under various abiotic stresses or external ABA treatment. By treating wild-type seedlings with exogenous CdCl_2_, NaCl, PEG, and ABA for 24 h, we evaluated the expression levels of all the *OsACO* genes by qRT-PCR (Figure 10).

Under CdCl_2_ treatment, the expression levels of *OsACO1*, *OsACO2,* and *OsACO7* were obviously reduced while other *OsACO* genes remained unaltered (Figure 10A). In response to salt stress, *OsACO5*, *OsACO6,* and *OsACO7* exhibited a dramatic decrease in expression whereas *OsACO1* increased by about 1.6 folds (Figure 10B). PEG-induced osmotic stress significantly inhibited the transcription of *OsACO1*, *OsACO2*, *OsACO3,* and *OsACO7* (Figure 10C). Exogenous ABA treatment also resulted in notable reductions in the expression of *OsACO5*, *OsACO6,* and *OsACO7* (Figure 10D). Overall, most *OsACO* genes exhibited suppressed transcription following 24 h of exposure to different abiotic stresses and ABA treatment.

## 3. Discussion

Ethylene plays crucial roles in various aspects of plant growth, development, and stress resilience [1,29]. ACO catalyzes the final step in the ethylene biosynthesis pathway, acting as the pivotal factor for the fine-tuned regulation of ethylene emission. In this study, we conducted a comprehensive analysis of the *OsACO* gene family by examining the evolutionary relationships, inter/intraspecies covariance, protein structures, conserved motifs, cis-acting elements, predicted miRNA targeting, potential post-translational modifications, and gene expression patterns across tissues and under abiotic stresses.

Seven putative *OsACO* genes, *OsACO1-7,* have been identified in the rice genome, classified into three subfamilies based on protein sequence similarity (Figure 1). However, direct evidence demonstrating the in vivo and in vitro activity of OsACO enzymes remains elusive. OsACOs belong to the Fe (II) ascorbate family of dioxygenase, characterized by nine highly conserved residues (indicated by black circles in Figure 5A). Iwai et al. (2006) presumed that OsACO4 and OsACO5 might be nonfunctional due to the absence of one or two of these conserved residues within motif 3 [18], but the functional significance of these missing residues and the enzyme activity of OsACO4 and OsACO5 are yet to be determined. Additionally, we noticed that OsACO7, the only member of Type II, lacks three conserved residues in motif 5 (Figure 5C), which are known to be critical for enzyme activity in other plant species [19,20]. OsACO7 also does not contain two motifs, motif 9 in Type I and motif 10 in Type III (Figure 5A), and its tertiary structure is the most divergent among OsACOs (except for OsACO6) (Figure 4). Therefore, further investigation is needed to elucidate the exact functionality of OsACO7.

Both our RT-qPCR results and RiceXPro transcriptome data revealed organ- and stage-specific expression patterns of the *OsACO* gene family (Figure 9), suggesting distinct regulatory roles for each member in rice growth and development. Similar expression patterns of *ACO* genes have also been documented in other plant species such as grapes [30]. Park et al. (2024) found that the induction of *OsACO4* in the embryo and endosperm can promote ethylene biosynthesis [31], ultimately facilitating rice seed germination (Nipponbare). This coincides with the transcriptome data showing a particularly high expression of *OsACO4* in these tissues at the reproductive stage (Figure 9B). We also identified a GCN4 motif uniquely present in the *OsACO4* promoter region, a cis-regulatory element involved in endosperm expression (Figure 6, Supplementary Table S4), implying that OsACO4 may play a specific role in seed development and germination. Yamauchi et al. (2016) reported that *OsACO5* predominantly contributed to the ethylene-mediated aerenchyma formation in aerated rice roots (Shiokari) [32], which aligned with our observation of a higher expression level of *OsACO5* in root tissue (Figure 9A,B). Additionally, Yamauchi et al. (2020) reported that the auxin regulation of aerenchyma formation is ethylene-dependent and involves the modulation of the *OsACO5* expression in rice roots (Taichung 65), further highlighting the potential importance of OsACO5 in root development [33]. We found that *OsACO1* transcripts mainly accumulated in both the stem and root in young rice seedlings (Figure 9A), which might be linked to its reported involvement in internode elongation (Nipponbare) and crown root primordium initiation (IR64) [34,35].

The transcriptional modulation of the *OsACO* gene family is a pivotal process for controlling ethylene biosynthesis in response to diverse external stimuli. For instance, we identified numerous cis-acting elements related to light response within *OsACO* promoters (Figure 6, Supplementary Table S4). Wang et al. (2016) observed that light can significantly induce OsACO protein accumulation [36], thereby promoting chlorophyll synthesis in rice (Japonica). OsPIL14 (Phytochrome-interacting factor-like14), a crucial modulator of light signaling, has been shown to directly bind to the promoter region of *OsACO1* and activates its transcription in rice (Nipponbare) [37]. Ethylene is essential for regulating drought tolerance in rice. It has been reported that drought can activate OsDERF1, a transcriptional repressor that suppresses *OsACO2* and *OsACO3* expression, resulting in reduced ethylene production and increased drought susceptibility at seedling stages (ZH11) [38]. In this study, exogenous treatment with 20% PEG-induced osmotic stress also significantly inhibited the expression of *OsACO2* and *OsACO3* (Figure 10C), linking these two genes to ethylene-mediated drought response. In addition, we observed a notable decrease in the expression of most *OsACO* genes under salinity (Figure 10B), contrasting with the increased OsACO enzyme activity and transcription under NaCl treatment reported by Siddikee et al. (Wuyunjing 7) [39]. It has been well established that plants undergo distinct stages in response to salt stress, including initial perception, osmotic adjustment, ion homeostasis, and transcriptional reprogramming, with dynamic gene expression patterns [40]. Therefore, our contradictory findings could be explained by differences in the timings of measurements (6 h vs. 24 h post exposure), suggesting the potential time-dependent alterations of *OsACO* expression in response to salt stress. Ethylene exerts complex influences on rice Cd tolerance, promoting resistance through ROS scavenging and apoplastic barrier formation (Nipponbare) [41] while also enhancing Cd absorption and accumulation via the activation of iron transporters (e.g., OSIRT1) [42]. We found that the expression of *OsACO1*, *OsACO2,* and *OsACO7* was repressed in CdCl_2_-treated seedlings (Figure 10A), suggesting that their downregulation may limit Cd uptake and alleviate toxicity by modulating ethylene biosynthesis.

Ethylene interacts with other plant hormones to regulate various biological processes in rice. For instance, ethylene has been reported to coordinate with auxin, ABA, GA, Br and CTK, etc. to regulate rice primary root growth through a complex network that influences root cell proliferation and elongation [43]. Growing evidence indicates the phytohormone-induced transcriptional regulation of *OsACO*s, further supported by the presence of cis-acting elements related to hormonal responses within their promoters (Figure 6, Supplementary Table S4). Chae et al. (2000) demonstrated that the transcription of *OsACO2* and *OsACO3* were differentially regulated by the crosstalk between auxin and ethylene [17]. Additionally, the transcription of *OsACO2* was found to be upregulated in response to exogenous cytokinin (CK) treatment (Nipponbare) [44]. ABA has been reported to antagonize ethylene in regulating rice growth. Lee et al. (2018) found that exogenous ABA treatment significantly downregulated the transcript levels of a subset of *OsACO* genes, leading to decreased ethylene synthesis in rice etiolated seedlings [45] (Nipponbare). Similarly, we also observed the remarkably inhibited expression of *OsACO5, OsACO6,* and *OsACO7* in the light-grown rice seedlings treated with external ABA (Figure 10B).

On the other hand, some evidence has shown that post-transcriptional regulation is also important for the transcript accumulation of *ACO* genes. In trifoliate orange, ptr-miR396b has been demonstrated to recognize and cleave *PtrACO*, leading to reduced ethylene synthesis [46]. In tomato, the miR164a-NAM3 module can directly regulate the expression of *SlACO1* and *SlACO4* [47]. However, information regarding this regulatory mechanism of *OsACO*s in rice is limited. By using psRNATarget, we have identified several putative targeting miRNAs for *OsACO3*, *OsACO4*, *OsACO5,* and *OsACO6* (Figure 7, Supplementary Table S5). Consistent with our prediction that osa-miR531b may target *OsACO4* (Figure 7), Park et al. (2024) observed an opposite expression pattern of this miRNA/target pair in the embryo during the same period after heading [31], with osa-miR531b downregulated and *OsACO4* induced, potentially facilitating ethylene production to promote rice seed maturation (Nipponbare). Baldrich et al. (2015) performed a combined analysis of miRNA and degradome profiles and found that an miR1846-guided regulation of *OsACO* in rice responds to fungal elicitors (Nipponbare) [48]. Further research is essential to elucidate the direct interactions between miRNAs and *OsACO*s, as well as their implications for ethylene biosynthesis in rice.

Post-translational modifications (PTMs) are crucial for ACO activity, but information on these processes remains largely unknown. Dilley et al. (2013) identified putative glycosylation and phosphorylation sites in apple MdACCO1 [20] and Ahmadizadeh et al. (2020) later computationally predicted similar sites for these two PTMs in rice OsACOs [49]. However, the impacts of these two PTMs on the stability and activity of OsACOs have yet to be clarified. In Arabidopsis, AtACO2 has been documented to be S-glutathionylated, despite the relevance of this modification to ACO stability/activity remaining unclear [50,51]. Two other types of thiol-residue modifications, *S*-nitrosylation (SNO) and *S*-sulfhydration (SSH), were observed in the ACOs of Arabidopsis and tomato [24,25,26,27,28]. The SSH modification of SlACO1 and SlACO2 showed inhibited activity [24] while the SNO modification of SlACO4h led to enzymatic activation [28]. Given these two modifications are evidenced by their direct effects on ACO activity, we predicted potential SSH and SNO sites for OsACOs (Figure 8, Supplementary Table S6), which may provide insights into the post-translational regulation of OsACOs in rice.

## 4. Materials and Methods

### 4.1. Database Search and Sequence Retrieval

The genomic sequences of the seven rice *OsACO* genes were obtained from the Rice Genome Annotation Project (https://rice.uga.edu/ (accessed on 13 June 2024)), including OsACO1 (LOC_Os09g27820), OsACO2 (LOC_Os09g27750), OsACO3 (LOC_Os02g53180), OsACO4 (LOC_Os11g08380), OsACO5 (LOC_Os05g05680), OsACO6 (LOC_Os05g05670), and OsACO7 (LOC_Os01g39860). All *OsACO* genes have a single transcript type, except for *OsACO3*, which has three different splice forms. Based on the work of Chae et al. [17], which involved isolating the cDNA clone of *OsACO3* using a highly conserved sequence of ACO enzyme family, corresponding to the splice form LOC_Os02g53180.2 (accession number AK071557), we selected this splice variant for further analysis. The genome databases and related annotation files for other plant species were downloaded from Phytozome 13 (https://phytozome-next.jgi.doe.gov/ (accessed on 2 July 2024)), including Arabidopsis (*Arabidopsis thaliana TAIR10*), wheat (*Triticum aestivum v2.2*), tomato (*Solanum lycopersicum ITAG5.0*), maize (*Zea mays RefGen_V4*), and barley (*Hordeum vulgare Morex V3*).

### 4.2. Phylogenetic Analysis and Classification of OsACOs

To investigate the phylogenetic relationships of ACOs in rice and other plant species, we utilized the full-length ACO protein sequences from *Oryza sativa* (Os), *Arabidopsis thaliana* (At), *Solanum lycopersicum* (Sl), *Zea mays* (Zm), *Hordeum vulgare* (Hv), *Malus domestica* (Md), and *Triticum aestivum* (Ta). We aligned the multiple amino acid sequences by using the ClustalW method in MEGA11, and then constructed the phylogenetic tree using the neighbor-joining method, with the following parameters: bootstrap repetition of 1000 times, Poisson pattern model, and pairwise deletions for gap handling. The phylogenetic tree was further formatted by iTOL V6 (https://itol.embl.de/ (accessed on 29 July 2024)). The seven OsACO proteins were categorized into three clades (Type I-III) based on the established classification for AtACOs in Arabidopsis.

### 4.3. Analysis of Chromosomal Distribution, Collinearity, and Selection Pressure in the OsACO Gene Family

The location information on seven *OsACO* genes on the chromosomes was obtained from the rice genome annotation file and visualized using TBtools [52]. To investigate the collinearity of *OsACO* genes with other plant species, we employed the One Step McScanX program in TBtools to generate collinear files and highlighted the homologous gene pairs between species using Dual Synteny Plot function. In addition, tandem duplications and collinearity within the rice genome were also assessed by McScanX, with the intraspecific collinear visualized using Advanced Circos feature in TBtools. We also evaluated the molecular evolutionary rates for each syntenic gene pair by using the Simple Ka/Ks Calculator (NG) program in TBtools.

### 4.4. Secondary and Tertiary Structural Prediction of OsACO Proteins

The secondary structures of OsACO proteins were analyzed by SOPMA online tool (https://npsa.lyon.inserm.fr/cgi-bin/npsa_automat.pl?page=/NPSA/npsa_sopma.html (accessed on 8 June 2024)). The prediction was set to classify the structures into four different conformational states, including α-helix, extended strand, β-turn, and random coil. The 3D structures of OsACO proteins were predicted using the AlphaFold2 platform (https://alphafold.ebi.ac.uk/ (accessed on 18 September 2024)) with the model confidence evaluated by the pLDDT score [53]. The tertiary structural similarity between each pair of OsACO proteins was assessed using the TM-score (http://zhanggroup.org/TM-score/ (accessed on 12 September 2024)) [54,55].

### 4.5. Analysis of Conserved Motifs in OsACO Proteins

The conserved motifs of the seven OsACO proteins were predicted using MEME (https://meme-suite.org/ (accessed on 17 August 2024)) with maximum width of 50 and minimum width of 6 and the detailed schematic distribution of eight conserved motifs was visualized by TBtools. The protein sequences of OsACO family members were aligned using Jalview (https://www.jalview.org/ (accessed on 11 August 2024)) with the MUSCLE algorithm with default settings and sorted by the MuscleWS ordering. Domain information for each motif was obtained from Pfam database (hosted by InterPro, https://www.ebi.ac.uk/interpro/ (accessed on 12 September 2024)).

### 4.6. Cis-Acting Element Analysis of OsACO Gene Promoters

The promoter sequence ranging from 2559 bp to 4439 bp upstream of start codon for each *OsACO* gene was extracted by TBtools and submitted to PlantCare website (http://bioinformatics.psb.ugent.be/webtools/plantcare/html (accessed on 16 September 2024)) for prediction of cis-regulatory elements related to plant growth, development, hormone response, and stress response. The results were visualized by using the R program (R scripts provided in File S1).

### 4.7. Putative microRNA Target Site Analysis

The cDNA sequences of all seven *OsACO* genes were submitted to the psRNA Target website (https://www.zhaolab.org/psRNATarget/analysis?function=2 (accessed on 20 September 2024)) to search for the potential miRNA target sites with maximum expectation threshold of ≤3.0 [56]. The predicted miRNAs and their corresponding target *OsACO* genes were visualized by using Cytoscape v3.10.2 software with the Attribute Grid layout.

### 4.8. Expression Profile Analysis of OsACO Genes

Based on the published transcriptome data from the RiceXPro database (https://ricexpro.dna.affrc.go.jp/ (accessed on 13 June 2024)), we retrieved the transcript levels of all seven *OsACO* genes across various tissues of wild type (Nipponbare) during the reproductive stages, including root (76 days after transplanting, DAT), leaf sheath (76 DAT), leaf blade (76 DAT), stem (before heading), anther (1.6–2.0 mm), inflorescence (5.0–10.0 mm), pistil (from 14–18 cm inflorescence), ovary (7 days after flowering, DAF), embryo (42 DAF), endosperm (42 DAF), palea (from 7.0 mm floret), and lemma (from 7.0 mm floret). A heatmap illustrating the expression patterns of the *OsACO* gene family in these tissues was generated by TBtools.

### 4.9. Plant Material and Exogenous Treatments

Wild-type rice seeds (ZH11) were soaked in 75% ethanol for 1 min, followed by immersion in 1.5% sodium hypochlorite solution for 30 min with shaking (80 rpm/min). The seeds were rinsed with distilled water for 5 times and then soaked in distilled water at 4 °C overnight in the dark. Afterwards, the seeds were placed on sterilized moist gauze for germination at 26 °C for 48 h. The germinated seeds were grown for 14 days under a 16 h light/8 h dark cycle at 26 °C and 60% humidity and the seedlings with uniform growth were selected for further gene expression assay. For abiotic stress treatments, 14-day-old seedlings were transferred to a hydroponic medium containing 50 µM ABA, 50 µM CdCl_2_, 150 mM NaCl, or 20% PEG 6000. After 24 h of exposure, samples were frozen in liquid nitrogen and stored at −80 °C for further RNA extraction.

### 4.10. Real-Time Quantitative PCR (RT-qPCR) Analysis

Fourteen-day-old rice seedlings were separated into three tissue types: root, stem, and leaf. For each tissue type, samples collected from four independent seedlings were pooled to create one biological replicate and a total of four bio-replicates were prepared for each group. For the abiotic stress assay, 14-day-old rice seedlings were treated with various conditions for 24 h, and three whole seedlings were mixed as one biological replicate, with a total of four bio-replicates prepared for each treatment group.

Total RNA was isolated using the Eastep^®^ Super Total RNA Extraction Kit (Promega, Madison, WI, USA), and the cDNA was synthesized using the Hifair^®^ III 1st Strand cDNA Synthesis for qPCR (gDNA digester plus) Kit (YEASEN, Shanghai, China). RT-qPCR was conducted on LineGene 9600 Plus system (Bioer, Hangzhou, China) using Hieff^®^ qPCR SYBR Green Master Mix (No Rox) kit (YEASEN). The relative expression levels were calculated using the 2^−ΔΔCT^ method with *OsUBQ* (LOC_Os01g22490) as an internal reference gene [57]. The bar graphs and statistical analyses were performed using Prism software (version 8.0.1, GraphPad, San Diego, CA, USA). The primer sequences used for RT-qPCR were designed by PRIMER PREMIER 5.0 and have been listed in Supplementary Table S7.

### 4.11. The Predicted Protein Modifications of OsACOs

The S-nitrosylation (SNO) and S-sulfhydration (SSH) of cysteine residues in OsACOs were predicted using the online tool pCysMod (http://pcysmod.omicsbio.info/ (accessed on 21 September 2024)) [58].

## 5. Conclusions

In the current study, seven *OsACO* genes were grouped into three subfamilies based on phylogenetic analysis, with members in the same cluster sharing similar structures and conserved motifs. This analysis provided clues as to the critical structures and residues essential for enzyme activity, informing the future functional characterization of OsACO proteins. Consistent with numerous cis-acting elements identified in the promoter regions of *OsACO*s that respond to development, stresses, hormones, and light, we observed the tissue- and developmental-specific expression patterns of *OsACO*s along with their alterations in response to abiotic stresses and ABA treatment. These findings underscore the potential role of the transcriptional regulation of *OsACO*s involved in rice growth and stress resilience. Furthermore, the prediction of putative targeting microRNAs and candidate cysteine sites of SNO and SSH in OsACOs offer additional insights into their post-transcriptional and post-translational regulatory mechanisms. Our findings not only offer essential information for the further experimental analysis of OsACO enzyme activity and regulation at multiple levels but also lay the foundation for developing strategies to enhance rice yield and stress tolerance by modulating ethylene biosynthesis via OsACOs.

## Figures and Tables

**Table 1:** Secondary structure analysis of OsACO proteins.

Gene ID	α-Helix (%)	Extended Strand (%)	β-Turn (%)	Random Coil (%)
OsACO1	38.82	16.46	5.59	39.13
OsACO2	36.65	16.77	5.9	40.68
OsACO3	39.88	16.20	5.61	38.32
OsACO4	36.89	17.80	7.12	38.19
OsACO5	34.09	16.88	6.17	42.86
OsACO6	32.48	21.66	10.19	35.67
OsACO7	37.50	16.67	5.45	40.38

## Data Availability

The data presented in this study are available in the paper and Supplementary Files.

## Supplementary Materials

The following supporting information can be downloaded at: https://www.mdpi.com/article/10.3390/plants13243490/s1, Table S1: The evolutionary constraint (Ka/Ks) between each syntenic gene pair. Table S2: Secondary structures of OsACO proteins. Table S3: The sequences of conserved motifs in OsACO proteins. Table S4: Predicted cis-regulatory elements in the promoter regions of *OsACO* genes. Table S5: Predicted miRNA-targeting *OsACO* mRNAs. Table S6: Putative amino acid modifications of OsACOs in rice. Table S7: Primer sequences used for RT-qPCR. The scripts used in R program for visualizing the results of cis-elements analysis have been provided in File S1.
