# Supplementary material for: Genome-Wide In Silico Analysis of 1-Aminocyclopropane-1-carboxylate oxidase (ACO) Gene Family in Rice (Oryza sativa L.)
Source: Plants (Basel). 2024 Dec 13;13(24):3490. doi: 10.3390/plants13243490 (PMC11728468; doi:10.3390/plants13243490)
Supplement: Supplementary file 1 [file plants-13-03490-s001.zip › File S1.pdf]

```r
# The R version used in this study is 4.4.0.
# Install the required R packages in advance
library(tidyverse)
library(ggplot2)
library(xlsx)

setwd("D:/R-4.4.0/OsACO")
df <- read_csv("D:/R-4.4.0/OsACO/cis.classification.tab.csv",col_names = F)
# Open the processed PlantCARE file; ensure the file is in CSV format to avoid potential errors.
# Sort by the third column
df <- df %>% arrange(X3)
print(df)

# Remove duplicates from the second and third columns and reset factor levels

uniq_level <- df %>% distinct(X2,X3)
df$X2 <- factor(df$X2, levels = uniq_level$X2)

# Clean the data: group by the first column X1 and the second column X2, count the number of occurrences in each group, sort by count (descending), and assign the result to tidy
tidy <- df %>%
  group_by(X1,X2) %>%
  summarise(number = n()) %>%
  arrange(desc(number))

# Read the sorting order from the file (subfamily grouping of gene families)
order <- readLines("order.txt")
tidy$X1 <- factor(tidy$X1, levels = order)
tidy <- tidy[order(tidy$X1), ]

low <- rgb(255,240,235,max = 255)

# Create a statistical plot using ggplot
ggplot(tidy, aes(x = X2, y = X1, fill = number)) +
  geom_tile(color = 'black') +
  geom_text(aes(label = number),col='black',cex = 3, size = 13) +
  scale_fill_gradient(low = low, high = "red") +
  scale_x_discrete(position = "top")+
  theme_classic() +
  theme(
    legend.title = element_blank(),
    legend.position = "bottom",
```

```
        axis.ticks = element_blank(),
        axis.line = element_blank(),
        axis.text.x = element_text(angle = 90, hjust = 0),
        axis.title = element_blank(),
        axis.text = element_text(size = 13, color = 'black')
    )
ggsave("cis_acting_element-1.svg",device = "svg",width = 18,height = 13)
```
